# Supplementary material for: A set of multi-entry identification keys to African frugivorous flies (Diptera, Tephritidae)
Source: Zookeys. 2014 Jul 24;(428):97–108. doi: 10.3897/zookeys.428.7366 (PMC4143993; doi:10.3897/zookeys.428.7366)
Supplement: Supplementary material 6 — Key to Ceratitis [file zookeys-428-097-s006.zip › SF6_ZooKeys_key to Ceratitis/key/SF6_ZooKeys_key to Ceratitis/Media/Html/Ceratitis semipunctata .htm]

Ceratitis (Pardalaspis) semipunctata sp


***Ceratitis*** **(*Pardalaspis*)
*semipunctata* De Meyer**

*Ceratitis* (*Pardalaspis*)
*semipunctata* De Meyer, 1996 : 24.

Body
length: 7.25 (7.0-7.5) mm; wing length: 7.5 mm.

Female

Head.
Antennal segments orange. Third antennal segment twice to three times as long
as second segment. Arista basal part pale, otherwise dark. Frons pale yellowish
in ground color, silvery over entire length; with dispersed short hairs,
distinctly darker than frons. Ocellar triangle dark. Face pale yellow. Occiput
moderately swollen below, pale whitish. Chaetotaxy normal for subgenus.

Thorax.
Ground colour of mesonotum pale; longitudinal lines hardly discernible.
Postpronotum same colour as mesonotum. Chaetotaxy normal for subgenus.
Mesonotum with pale pilosity. Anepisternum along upper margin with darker hair,
and a few dark hairs on lower part of anepisternum. Two or three anepisternal
bristles.� Scutellum yellow with apical markings black; basally without dark
spots, only a faint darker coloration.

Legs
pale yellow; setation typical of subgenus. Front femur predominantly with pale
setae, posterior row dark.

Wings
with brownish bands, yellow markings strongly reduced. Banding, setation and
venation normal for subgenus. Marginal band continuous; discal band joined with
marginal band; cross-vein r-m at middle of discal cell; vein R_1_
ending before cross-vein r-m.

Abdomen.
Greyish, with clearly defined spots. Pattern of spots and setation normal for
subgenus.� Oviscape shorter than abdominal terga 3-6, yellow-orange in colour.

Male

Unknown.

(Description
after De Meyer, 1996)
